# Antifeedant Activities of Lignans from Stem Bark of *Zanthoxylum armatum* DC. against *Tribolium castaneum*

**DOI:** 10.3390/molecules23030617

**Published:** 2018-03-09

**Authors:** Wenjuan Zhang, Yang Wang, Zhufeng Geng, Shanshan Guo, Juqin Cao, Zhe Zhang, Xue Pang, Zhenyang Chen, Shushan Du, Zhiwei Deng

**Affiliations:** 1 Beijing Key Laboratory of Traditional Chinese Medicine Protection and Utilization, Faculty of Geographical Science, Beijing Normal University, No. 19 Xinjiekouwai Street, Beijing 100875, China; zwj0729@mail.bnu.edu.cn (W.Z.); wangyangjs@mail.bnu.edu.cn (Y.W.); gengzhufeng@bnu.edu.cn (Z.G.); guoshanshan@mail.bnu.edu.cn (S.G.); juqincao@mail.bnu.edu.cn (J.C.); zhezhang@mail.bnu.edu.cn (Z.Z.); pangxue0924@mail.bnu.edu.cn (X.P.); sunnychen@mail.bnu.edu.cn (Z.C.); 2 Analytical and Testing Center, Beijing Normal University, No. 19 Xinjiekouwai Street, Haidian District, Beijing 100875, China; dengzw@bnu.edu.cn

**Keywords:** *Zanthoxylum armatum*, *Tribolium castaneum*, antifeedant activity, chemical constituents, methylenedioxy

## Abstract

The speciation of a methanolic extract of *Zanthoxylum armatum* stem bark has enabled the isolation and characterization of 11 known lignans. Among them, five compounds (**6**, **8**–**11**) are reported in this plant for the first time. All of the chemical structures were elucidated on the basis of NMR spectral analysis. Additionally, their antifeedant activities against *Tribolium castaneum* were evaluated scientifically. Among them, asarinin (**1**), with an EC_50_ of 25.64 ppm, exhibited a much stronger antifeedant activity than the positive control, toosendanin (EC_50_ = 71.69 ppm). Moreover, fargesin (**2**), horsfieldin (**3**), and magnolone (**10**), with EC_50_ values of 63.24, 68.39, and 78.37 ppm, showed almost the same antifeedant activity as the positive control. From the perspective of structure-effectiveness relationship, compounds with the chemical group of methylenedioxy exhibited higher antifeedant activities and have potential to be developed into novel antifeedants or potential lead compounds to protect food and crops in storage.

## 1. Introduction

*Tribolium castaneum*, the red flour beetle, is one of the most destructive storage pests and has a direct effect on the reduction of the quantity and quality of grain during postharvest storage [1]. According to reports, the damages to stored grains and their related grain products by storage pests could be as high as 20–30% in tropical countries [2]. As an invertebrate model organism, *T. castaneum* is used in various biological disciplines, including developmental biology, immunology, evolutionary studies, and ecology [3,4,5]. Nowadays, synthetic insecticides have been commonly and heavily used to protect stored grains and their related products against pests. However, massive and repeated usage of synthetic insecticides has resulted in a series of problems, including pesticide residue, environmental pollution, and insect resistance [6,7]. Therefore, increased attention has been given to develop alternative strategies which include using plant-derived compounds as effective natural anti-insect agents or leading compounds for the control of insects [6,8]. Plants containing bioactive compounds could provide an alternative source of insect control agents and many of them are selective and have little or no harmful effects on the environment and non-targeted organisms [9,10].

*Zanthoxylum armatum* DC., belonging to the family Rutaceae, is a traditional evergreen shrub that is distributed in Southeast Asia [11]. In China, it is widely cultivated in the South of China, especially in Sichuan province [12]. As a Chinese traditional folk medicinal herb, *Z. armatum* is applied for toothaches, stomachaches, and wet sores [13]. According to reports, the chemical composition category of *Z. armatum* is various, and including alkaloids, saponins, phenolic glycosides, coumarins, lignans, sterols, flavonoids, and terpenes [14,15,16,17,18,19]. Different chemical constituents result in different bioactivities, such as larvicidal [18], insecticidal [20], antioxidant [21,22], antibacterial [23], cytotoxic [24,25], antimicrobial [11], and anthelmintic [26] activities. In our present screening program, 11 lignans were isolated from *Zanthoxylum armatum* and some of them showed significant antifeedant activity. Moreover, their antifeedant activities were evaluated for the first time.

## 2. Results

**Figure 1:**
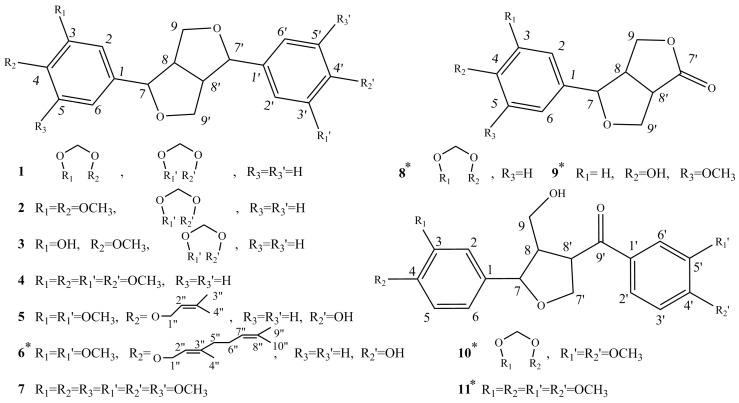
Chemical structures of compounds **1**–**11**. * represents known but derived from the plant of *Zanthoxylum armatum* for the first time.

In total, 11 known lignans, asarinin (**1**), fargesin (**2**), horsfieldin (**3**), eudesmin (**4**), planispine A (**5**), planispine B (**6**), yangambin (**7**), acuminatolide (**8**), salicifoliol (**9**), magnolone (**10**), and magnone A (**11**), were isolated from the methanol extract of *Z. armatum* through a variety of column chromatography and preparative HPLC processes. Their chemical structures are displayed in Figure 1. Among them, five compounds, planispine B (**6**), acuminatolide (**8**), salicifoliol (**9**), magnolone (**10**), and magnone A (**11**), were isolated from this plant for first time. The structural characteristics of the 11 lignans are obvious. As shown in Figure 1, compounds **1**–**7**, compounds **8**–**9**, and compounds **10**–**11** each have the same basic mother nucleus. Compared with compounds **1**–**7**, the basic mother nucleus of compounds **8**–**9** only has one benzene cycle on one side, while for compounds **8**–**9**, one of the furan cycles on the mother nucleus is opened. Therefore, different chemical structures with different substituents may result in different bioactivities.

**Figure 2:**
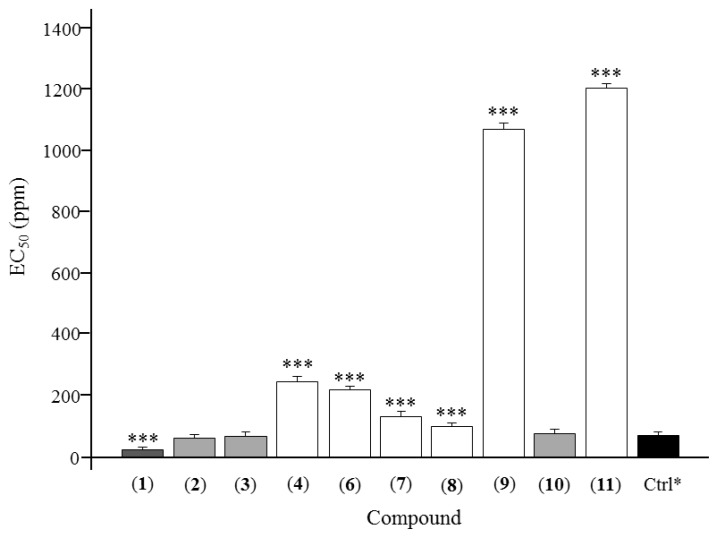
Antifeedant EC_50_ of 10 lignans from *Zanthoxylum armatum.* * represents the positive control, toosendanin. *** represents the significant difference at *p* < 0.05.

The antifeedant activities of the 11 lignans displayed in Figure 1 were measured, and the results are presented in Table 1. At the lowest concentration of 25 ppm, asarinin (**1**) exhibited the highest antifeedant activity against *T. castaneum* with an antifeedant index of 49.45%. Furthermore, fargesin (**2**), acuminatolide (**8**), magnolone (**10**), yangambin (**7**), eudesmin (**4**), and horsfieldin (**3**) also showed higher antifeedant activities against *T. castaneum* than the positive control, toosendanin, at the lowest concentration of 25 ppm, with antifeedant indices of 40.65%, 39.15%, 38.93%, 37.80%, 35.73%, and 34.99%, respectively. The EC_50_ of 10 lignans are displayed in Figure 2. As shown in Figure 2, eudesmin (**4**), planispine B (**6**), yangambin (**7**), acuminatolide (**8**), salicifoliol (**9**), and magnone A (**11**) exhibited significant differences compared to the positive control, toosendanin, at *p* < 0.05. Meanwhile, fargesin (**2**), horsfieldin (**3**), and magnolone (**10**) showed nearly the same antifeedant level against *T. castaneum*. Asarinin (**1**) was the only compound that exhibited a lower EC_50_ than the positive control with the significant difference level at *p* < 0.05.

## 3. Discussion

Compared with traditional isolation method, the application of preparative HPLC provided an effective way to achieve the isolation and purification of compounds. Moreover, this method provides a basis for the establishment of the method of compound separation and purification. After that, 11 compounds were identified as lignan compounds. As reported in the literature [27], lignans are a large group of natural products, and many of them exhibit potential bioactivities with antioxidant, antitumor, anti-inflammatory, and antiviral properties. Here, the antifeedant activities of lignans isolated from *Z. armatum* were investigated.

As shown in Table 1, there was a dose-dependent antifeedant activity, and the antifeedant activity increased with the increase of the concentration gradient from 25 ppm to 2000 ppm. Most of the lignans exhibited antifeedant activities against *T. castaneum*. At a concentration of 74 ppm, asarinin (**1**) and fargesin (**2**) showed high antifeedant activities with an antifeedant index above 50%. At a concentration of 222 ppm, asarinin (**1**), fargesin (**2**), horsfieldin (**3**), and magnolone (**10**) exhibited higher antifeedant activities than other lignans. Moreover, asarinin (**1**) and fargesin (**2**) showed nearly the same antifeedant index as the positive control, toosendanin, at a concentration of 2000 ppm. According to the antifeedant results at different concentrations, asarinin (**1**) is the most active compound, which has potential to be developed into novel antifeedant formulations for insect control in storage. In Figure 2, asarinin (**1**), with an EC_50_ of 25.64 ppm, exhibited a significant difference compared to the positive control, toosendanin (EC_50_ = 71.69 ppm) at *p* < 0.05. What is more, fargesin (**2**), horsfieldin (**3**), and magnolone (**10**) showed the same antifeedant activity as the positive control, with EC_50_ values of 63.24, 68.39, and 78.37 ppm, respectively. Meanwhile, eudesmin (**4**), planispine B (**6**), yangambin (**7**), and acuminatolide (**8**), with EC_50_ values of 245.72, 219.84, 132.50, and 102.12 ppm, respectively, showed less antifeedant activity compared to the positive control at *p* < 0.05. Salicifoliol (**9**) (EC_50_ = 1069.20 ppm) and magnone A (**11**) (EC_50_ = 1203.53 ppm) exhibited much less antifeedant activity, and planispine A (**5**) showed no antifeedant activity. From the perspective of structure-effectiveness relationship, asarinin (**1**), having two methylenedioxy groups, showed the highest antifeedant activity. Moreover, fargesin (**2**), horsfieldin (**3**), magnolone (**10**), and acuminatolide (**8**), each having one group of methylenedioxy, displayed less antifeedant activity than asarinin (**1**). Salicifoliol (**9**), magnone A (**11**), and planispine A (**5**), having no methylenedioxy groups, showed much less or no antifeedant activity. As it was reported, compounds with methylenedioxy groups have various bioactivities, such as improving skin condition, antioxidant activity [28], anti-inflammatory activity [29], radical scavenging activity [30], insecticidal activity [31], antineuroinflammatory effects [32], antiparasitic activity, and anti-osteoporotic activity [33]. Therefore, methylenedioxy, as an active chemical group, may play a key role in antifeedant activities. Moreover, compounds with one or more methylenedioxy groups might be developed into potential lead compounds or conventional antifeedant alternatives. Since the natural resources of *Z. armatum* are abundant, it is meaningful to take advantage of its non-medicinal parts to achieve a comprehensive utilization of resources.

## 4. Materials and Methods

### 4.1. General Information

NMR spectra were recorded on a Bruker Avance III 500 instrument (Bruker-Biospin, Billerica, MA, USA) at room temperature. Preparative HPLC was performed on a Waters Delta Prep 4000 system, which was equipped with a Waters 2487 dual λ absorbance detector. A Rainbow Kromasil-C_18_ (10 mm × 250 mm, 10 μm) column was selected and performed for preparative HPLC. Column chromatography was performed on silica gel (160–200 mesh) and Thin Layer Chromatography (TLC) analysis was carried out on silica gel G plates (Qingdao Marine Chemical Plant, Qingdao, China). Sephadex LH-20 was purchase from Amersham Pharmacia Biotech (Beijing, China). Mitsubishi Chemical Ion (MCI) gel CHP20P (75–150 μm) was supplied by Kaiteki Company (Tokyo, Japan). All of the analytical reagents were purchased from Beijing Chemical Factory (Beijing, China). The deuterated solvents (CDCl_3_, Deuterated ratio, 99.8%) with tetramethylsilane (TMS) were purchased from Cambridge Isotope Laboratories, Inc. (Andover, MA, USA).

### 4.2. Material

#### 4.2.1. Plants

The fresh stem bark of *Z. armatum* was collected in Wen county, Gansu Province, China (32.95 N latitude, 104.70° E longitude) on 2 September 2015. The plant was identified by Dr. Liu, Q.R. (College of Life Sciences, Beijing Normal University, Beijing, China) and the voucher specimen (BNU-CMH-DuSS-2015-09-02-001) was deposited at the Herbarium (BNU) of the Faculty of Geographical Science, Beijing Normal University.

#### 4.2.2. Insects

The red flour beetle, *Tribolium castaneum*, was identified by Liu, Z.L. (College of Plant Protection, China Agricultural University, Beijing, China). A laboratory culture of *T. castaneum* followed the same method mentioned in the recent literature [34]. The red flour beetle was maintained in the dark cabinet of an incubator at 29–30 °C and 70–80% relative humidity (RH). Glass containers (0.5 L) containing wheat flour at 12–13% moisture content mixed with yeast (10:1, *w*/*w*) were used for insect culture. The unsexed insect adults used in the experiments were about 7 days old.

### 4.3. Extraction and Isolation

**Figure 3:**
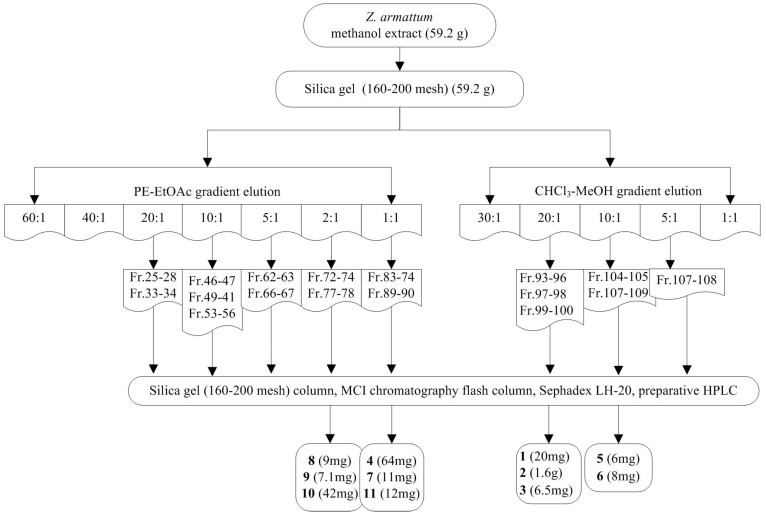
Chemical isolation scheme of *Zanthoxylum armatum* methanol extract.

The fresh stem bark (1.6 kg) of *Z. armatum* was roughly crushed and extracted with methanol (20 L) under ultrasound three times. The filtrate was concentrated into crude extract (59.2 g) under vacuum. The extract was crudely fractionated by silica gel column chromatography (160–200 mesh, 10.0 × 63 cm, 1300 g), eluting with a stepwise gradient of PE (petroleum ether)/EtOAc (ethyl acetate) (60:1, 40:1, 20:1, 10:1, 5:1, 2:1 and 1:1), and then CHCl_3_/CH_3_OH (30:1, 20:1, 10:1, 5:1, 1:1 and MeOH) to receive 108 fractions. In total, 11 lignans were isolated from the stem bark of *Z. armatum*. The chemical isolation scheme of *Z. armatum* methanol extract is shown in Figure 3. All purified products were analyzed by ^1^H and ^13^C-NMR spectra and then compared with literature values. Accordingly, lignans **1**–**11** were characterized and identified as asarinin (**1**) [35], fargesin (**2**) [31], horsfieldin (**3**) [36], eudesmin (**4**) [37], planispine A (**5**) [36], planispine B (**6**) [38], yangambin (**7**) [39], acuminatolide (**8**) [40], salicifoliol (**9**) [41], magnolone (**10**) [42], and magnone A (**11**) [43].

Asarinin (**1**). White powder. ^1^H-NMR (500 MHz, CDCl_3_) *δ* ppm: 6.89 (2H, s, H-2,2′), 6.85~6.79 (4H, m, H-5,5′,6,6′), 5.98 (2H, s, -O-CH_2_-O-), 5.97 (2H, s, -O-CH_2_-O-), 4.85 (1H, s, H-7′), 4.42 (1H, s, 7.0 Hz, H-7), 4.12 (1H, s, H-9′α), 3.85 (2H, m, H-9α,9′β), 3.32 (2H, m, H-9β,8′), 2.88 (1H, m, H-8); ^13^C-NMR (500 MHz, CDCl_3_) *δ* ppm: 147.9 (C-4′), 147.6 (C-4), 147.2 (C-3), 146.6 (C-3′), 135.1 (C-1), 132.3 (C-1′), 119.6 (C-6), 118.7 (C-6′), 108.2 (C-5′), 106.6 (C-2′), 106.4 (C-2), 101.1 (-O-CH_2_-O-), 101.0 (-O-CH_2_-O-), 87.7 (C-7), 82.0 (C-7′), 70.9 (C-9′), 69.9 (C-9), 54.7 (C-8), 50.2 (C-8′).

Fargesin (**2**). Colorless cluster crystals. ^1^H-NMR (500 MHz, CDCl_3_) *δ* ppm: 6.95 (1H, s, H-2), 6.90 (1H, s, H-2′), 6.88 (2H, s, H-5, 6), 6.85 (1H, d, *J* = 8.0 Hz, H-6′), 6.80 (1H, d, *J* = 8.0 Hz, H-5′), 5.97 (2H, s, -OCH_2_O-), 4.89 (1H, d, *J* = 5.0 Hz, H-7), 4.44 (1H, d, *J* = 7.0 Hz, H-7′), 4.14 (1H, d, *J* = 9.5 Hz, H-9′α), 3.93 (3H, s, 3-OCH_3_), 3.90 (3H, s, 4-OCH_3_), 3.86 (2H, dd, *J* = 8.0 Hz, *J* = 7.0 Hz, H-9α, 9′β), 3.44 (2H, m, H-9β, 8), 2.90 (1H, dd, *J* = 8.0 Hz, H-8′); ^13^C-NMR (125 MHz,CDCl_3_) *δ* ppm: 148.8 (C-3), 148.0 (C-4), 148.0 (C-3′), 147.2 (C-4′), 135.2 (C-1′), 130.9 (C-1), 119.6 (C-6′), 117.7 (C-6), 111.0 (C-5), 109.0 (C-2), 108.2 (C-5′), 106.5 (C-2′), 101.1 (-OCH_2_O-), 87.7 (C-7′), 82.0 (C-7), 71.0 (C-9′), 69.8 (C-9), 55.9 (3-OCH_3_), 55.9 (4-OCH_3_), 54.6 (C-8′), 50.2 (C-8).

Horsfieldin (**3**). Colorless needle crystals. ^1^H-NMR (500 MHz, CDCl_3_) *δ* ppm: 6.97 (1H, s, H-2), 6.91 (1H, d, *J* = 8.0 Hz, H-2′), 6.90 (1H, s, H-5), 6.85 (1H, d, *J* = 8.0 Hz, H-5′), 6.80 (2H, d, *J* = 8.0 Hz, H-6,6′), 5.98 (2H, s, -OCH_2_O-), 5.60 (1H, s, 3-OH), 4.87 (1H, d, *J* = 5.0 Hz, H-7′), 4.44 (1H, d, *J* = 7.0 Hz, H-7), 4.14 (1H, d, *J* = 9.5 Hz, H-9′α), 3.94 (3H, s, 4-OCH_3_), 3.86 (2H, m, H-9β,9′β), 3.33 (2H, m, H-9α,8′), 2.89 (1H, m, H-8); ^13^C-NMR (125 MHz, CDCl_3_) *δ* ppm: 148.0 (C-3′), 147.2 (C-4), 146.4 (C-3), 144.6 (C-4′), 135.2 (C-1), 130.3 (C-1′), 119.6 (C-6), 118.4 (C-6′), 114.2 (C-2), 108.3 (C-5), 108.2 (C-5′), 106.6 (C-2′), 101.1 (-OCH_2_O-), 87.7 (C-7), 82.0 (C-7′), 71.0 (C-9′), 69.8 (C-9), 56.0 (4-OCH_3_), 54.6 (C-8), 50.2 (C-8′).

Eudesmin (**4**). Colorless cluster crystals. ^1^H-NMR (500 MHz, CDCl_3_) *δ* ppm: 6.93 (2H, s, H-2,2′), 6.90 (2H, d, *J* = 8.0 Hz, H-5,5′), 6.87 (2H, d, *J* = 8.0 Hz, H-6,6′), 4.78 (2H, t, *J* = 3.0 Hz, H-7,7′), 4.28 (2H, t, d, *J* = 8.0 Hz, H-9α,9′α), 3.92 (8H, s, 3,3′-OCH_3_,H-9β,9′β), 3.90 (6H, s, 4,4′-OCH_3_), 3.14 (2H, s, H-8,8′), ^13^C-NMR (125 MHz, CDCl_3_) *δ* ppm: 149.2 (C-3,3′), 148.6 (C-4,4′), 133.6 (C-1,1′), 118.3 (C-6,6′), 111.0 (C-2,2′), 109.2 (C-5,5′), 85.8 (C-7,7′), 71.7 (C-9,9′), 56.0 (C-3,3′), 55.9 (C-4,4′), 54.2 (C-8,8′).

Planispine A (**5**). Colorless oil. ^1^H-NMR (500 MHz, CDCl_3_) *δ* ppm: 6.98 (1H, s, H-6), 6.94 (1H, s, H-6′), 6.91 (1H, d, *J* = 8.5 Hz, H-5), 6.88 (2H, d, H-5′,2), 6.81 (1H, d, *J* = 8.0 Hz, H-2′), 5.61 (1H, s, 4′-OH), 5.54 (1H, t, *J* = 6.0 Hz, H-2″), 4.88 (1H, d, *J* = 5.0 Hz, H-7), 4.60 (2H, d, *J* = 6.5 Hz, H-1″), 4.46 (1H, d, *J* = 7.0 Hz, H-8), 4.15 (1H, d, *J* = 7.0 Hz, H-9α), 3.94 (3H, s, 3′-OCH_3_), 3.91 (3H, s, 3-OCH_3_), 3.87 (2H, t, *J* = 7.0 Hz, H-9β,9′β), 3.34 (2H, d, *J* = 5.0 Hz, H-8,9′α), 2.94 (1H, m, H-8′); ^13^C-NMR (125 MHz, CDCl_3_) *δ* ppm: 149.7 (C-3), 148.0 (C-4), 146.4 (C-3′), 144.6 (C-4′), 137.6 (C-1), 133.7 (C-1′), 130.4 (C-6), 120.0 (C-2″), 118.4 (C-6′), 118.4 (C-5), 114.2 (C-5′), 112.9 (C-2′), 109.3 (C-3″), 108.4 (C-2), 87.7 (C-7′), 82.1 (C-7), 71.0 (C-9), 69.7 (C-9′), 65.8 (C-1″), 56.0 (3′-OCH_3_), 55.9 (3-OCH_3_), 54.4 (C-8′), 50.2 (C-8), 25.8 (C-4″), 18.2 (C-5″).

Planispine B (**6**). Colorless oil. ^1^H-NMR (500 MHz, CDCl_3_) *δ* ppm: 6.92~6.86 (6H, m, H-2,2′,5,6,6′,5′), 5.53 (1H, t, *J* = 5.5 Hz, H-2″), 5.10 (1H, t, *J* = 7.0 Hz, H-7″), 4.77 (2H, dd, *J* = 4.5 Hz, *J* = 7.5 Hz, H-7,7′), 4.63 (1H, d, *J* = 4.5 Hz, H-1″), 4.28 (2H, t, *J* = 8.0 Hz, H-9′), 3.93 (3H, s, 3-OCH_3_), 3.91 (5H, s, 3′-OCH_3_, H-9), 3.14 (2H, m, H-8,8′), 2.13 (2H, t, *J* = 8.0 Hz, H-5″), 2.07 (2H, m, H-6″), 1.74 (3H, s, H-4″), 1.69 (3H, s, H-9″), 1.62 (3H, s, H-10″); ^13^C-NMR (125 MHz, CDCl_3_) *δ* ppm: 149.7 (C-3), 147.9 (C-4), 146.7 (C-3′), 145.3 (C-4′), 140.6 (C-3″), 133.5 (C-1′), 132.9(C-1), 131.8 (C-8″), 123.9 (C-7″), 119.8 (C-2″), 119.0 (C-6′), 118.2 (C-6), 114.3(C-5′), 113.0 (C-5), 109.5 (C-2′), 108.6 (C-2), 85.9 (C-7), 85.8 (C-7′), 71.7 (C-9,9′), 66.0 (C-1″), 56.0 (3,3′-OCH_3_), 54.2 (C-8), 54.1 (C-8′), 39.6 (C-5″), 26.3 (C-6″), 25.7 (C-9″), 17.7 (C-10″), 16.7 (C-4″).

Yangambin (**7**). Colorless oil. ^1^H-NMR (500 MHz, CDCl_3_) *δ* ppm: 6.59 (4H, s, H-2,2′,6,6′), 4.77 (2H, d, *J* = 4.0 Hz, H-1,1′), 4.33 (2H, dd, *J* = 9.0 Hz, *J* = 7.0 Hz, H-9α,9′α), 3.95 (2H, dd, *J* = 9.0 Hz, *J* = 3.5 Hz, H-9β,9′β), 3.90 (12H, s, 3,3′,5,5′-OCH_3_), 3.86 (6H, s, 4,4′-OCH_3_), 3.13 (2H, m, H-8,8′); ^13^C-NMR (125 MHz, CDCl_3_) *δ* ppm: 153.5 (C-3,3′,5,5′), 137.5 (C-4,4′), 136.7 (C-1,1′), 102.8 (C-2,2′,6,6′), 86.0 (C-7,7′), 72.0 (C-9,9′), 60.9 (4,4′-OCH_3_), 56.2 (3,3′,5,5′-OCH_3_), 54.4 (C-8,8′).

Acuminatolide (**8**). Colorless needle crystals. ^1^H-NMR (500 MHz, CDCl_3_) *δ* ppm: 6.86 (1H, s, H-6), 6.82 (1H, s, H-2), 6.81 (1H, s, H-3), 5.99 (2H, s, H-10), 4.62 (1H, d, *J* = 7.0 Hz, H-7), 4.51 (1H, m, H-9β), 4.38 (1H, d, *J* = 7.0 Hz, H-9′β), 4.35 (1H, m, H-9α), 4.21 (1H, dd, *J* = 3.5 Hz, *J* = 9.5 Hz, H-9′α), 3.45 (1H, td, *J* = 3.5 Hz, *J* = 9.5 Hz, H-8), 3.10 (1H, m, H-8′); ^13^C-NMR (125 MHz, CDCl_3_) *δ* ppm: 178.1 (C-7′), 148.3 (C-5), 147.8 (C-4), 132.8 (C-1), 119.6 (C-2), 108.4 (C-3), 106.4 (C-6), 101.3 (-OCH_2_O-), 86.1 (C-7), 70.1 (C-9), 69.8 (C-9′), 48.4 (C-8), 46.0 (C-8′).

Salicifoliol (**9**). Colorless oil. ^1^H-NMR (500 MHz, CDCl_3_) *δ* ppm: 6.93 (1H, d, *J* = 8.0 Hz, H-3), 6.90 (1H, s, H-6), 6.83 (1H, d, *J* = 8.0 Hz, H-2), 5.68 (1H, s, 4-OH), 4.64 (1H, d, *J* = 7.0 Hz, H-7), 4.52 (1H, dd, *J* = 9.0 Hz, *J* = 7.0 Hz, H-9β), 4.39 (1H, t, *J* = 9.0 Hz, H-8β), 4.35 (1H, d, *J* = 11.0 Hz, H-9α), 4.21 (1H, dd, *J* = 9.0 Hz, *J* = 3.5 Hz, H-8α), 3.93 (3H, s, 5-OCH_3_), 3.47 (1H, td, *J* = 9.0 Hz, *J* = 3.5 Hz, H-12), 3.14 (1H, m, H-11); ^13^C-NMR (125 MHz, CDCl_3_) *δ* ppm: 178.2 (C-10), 146.9 (C-5), 145.9 (C-4), 130.6 (C-1), 119.1 (C-2), 114.4 (C-3), 108.5 (C-6), 86.1 (C-7), 70.0 (C-8), 69.9 (C-9), 56.1 (5-OCH_3_), 48.2 (C-11), 46.0 (C-12).

Magnolone (**10**). White powder. ^1^H-NMR (500 MHz, CDCl_3_) *δ* ppm: 7.62 (1H, d, *J* = 8.5 Hz, H-6′), 7.59 (1H, s, H-2′), 6.99 (1H, s, H-2), 6.93 (d, 2H, *J* = 8.5 Hz, H-5′), 6.88 (1H, d, *J* = 8.0 Hz, H-6), 6.79 (1H, d, *J* = 8.0 Hz, H-5), 5.97 (2H, s, -OCH_2_O-), 4.70 (2H, d, *J* = 9.0 Hz, H-7), 4.31 (1H, m, H-9′β), 4.18 (1H, m, H-8′), 4.18 (1H, m, H-9′α), 3.98 (1H, s, 3′-OCH_3_), 3.96 (1H, s, 4′-OCH_3_), 3.79 (1H, dd, H-9β), 3.68 (1H, dd, H-9α), 2.88 (1H, m, H-8); ^13^C-NMR (125 MHz, CDCl_3_) *δ* ppm: 197.8 (C-7′), 153.7 (C-4′), 149.3 (C-3′), 148.0 (C-3), 147.4 (C-4), 134.5 (C-1), 129.8 (C-1′), 123.2 (C-6′), 120.4 (C-6), 110.6 (C-2′), 110.1 (C-5′), 108.1 (C-5), 107.1 (C-2), 101.1 (-OCH_2_O-), 83.7 (C-7), 70.9 (C-9′), 61.3 (C-9), 56.1 (3′-OCH_3_), 56.0 (4′-OCH_3_), 52.4 (C-8), 49.6 (C-8′).

Magnone A (**11**). Colorless oil. ^1^H-NMR (500 MHz, CDCl_3_) *δ* ppm: 7.63 (1H, d, *J* = 9.0 Hz, H-2′), 7.60 (1H, d, *J* = 1.5 Hz, H-6′), 7.05 (1H, d, *J* = 1.5 Hz, H-2), 6.95 (1H, d, *J* = 2.0 Hz, H-6), 6.93 (1H, d, *J* = 8.5 Hz, H-3′), 6.85 (1H, d, *J* = 8.0 Hz, H-5), 4.72 (1H, d, *J* = 9.0 Hz, H-7), 4.33 (1H, m, H-9′β), 4.21 (2H, m, H-8′,9′α), 3.98 (3H, s, 3-OCH_3_), 3.96 (3H, s, 4-OCH_3_), 3.94 (3H, s, 5′-OCH_3_), 3.89 (3H, s, 4′-OCH_3_), 3.80 (1H, dd, *J* = 11.0 Hz, *J* = 4.5 Hz, H-9β), 3.70 (1H, dd, *J* = 11.0 Hz, *J* = 4.5 Hz, H-9α), 2.94 (1H, m, H-8); ^13^C-NMR (125 MHz, CDCl_3_) δ ppm: 198.0 (C-7′), 153.7 (C-4′), 149.3 (C-5′), 149.2 (C-4), 148.9 (C-3), 133.0 (C-1), 129.8 (C-1′), 123.2 (C-2′), 119.3 (C-6), 110.9 (C-5), 110.6 (C-6′), 110.1 (C-3′), 109.6 (C-2), 83.8 (C-7), 70.9 (C-9′), 61.4 (C-9), 56.1 (3-OCH_3_), 56.0 (4/5′-OCH_3_), 55.9 (4′-OCH_3_), 52.2 (C-8), 49.7 (C-8′).

### 4.4. Antifeedant Assay

An antifeedant assay was performed using a modified method, as reported in References [44,45]. The compound sample was precisely weighed at 2 mg and diluted in ethanol to fabricate the mother liquor with a concentration of 1 mg/mL. Then, different volumes of the mother liquor were diluted in water to obtain different solution concentrations of 25, 74, 222, 667, and 2000 ppm. After that, the final solutions with different concentrations were stirred well with 0.4 g flour. The blank control was fabricated by the mixture of 2 mL water and 0.4 g flour. Then, 1-mL tips were cut about 1 cm from the bottom to make an opening, and enlarged to about a 2-mm internal diameter. Subsequently, 200 µL mixture was pipetted into a clean dish to make about 6–8 small cookies drop by drop. After being air-dried overnight, the small cookies with different concentrations were transferred into the incubator for 48 h to achieve equilibration at 29–30 °C and 70–80% relative humidity (RH). Twenty unsexed adult insects were selected and placed into glass vials (diameter: 2.5 cm, height: 5.5 cm) and starved for 24 h for further weighing. After that, small cookies were added into each vial and weighed later. For each concentration, the test was conducted five times. Seventy-two hours later, the total weight was determined. Then, all the insects were singled out immediately, and each vial with remaining cookies were weighed again. The percent antifeedant index was calculated as follows: Antifeedant index (%) = [(*C* − *T*)/*C*] × 100, where *C* is the weight of the diet consumed in the blank control and *T* is the weight of the diet consumed in the treated groups. The antifeedant index and EC_50_ were calculated by SPSS (IBM) and Origin software. Statistical analysis was carried out using Student’s *t*-test to compare the two groups, with *p* < 0.05 being indicative of significance. A commercial antifeedant reagent, toosendanin, used as a positive control, was purchased from China National Standards Network.

## 5. Conclusions

Eleven lignans were isolated from the stem bark of *Zanthoxylum armatum*, and five of them were obtained for the first time. The antifeedant activities of these compounds were measured, and one of them, asarinin (**1**), showed stronger activity than the positive control. Three other compounds, fargesin (**2**), horsfieldin (**3**), and magnolone (**10**), exhibited the same antifeedant activities as the positive control. Furthermore, compounds that displayed high antifeedant activities have a common chemical group, namely methylenedioxy. These results indicated that compounds with one or more methylenedioxy groups have potential for development into novel antifeedants or lead compounds to protect food crops in storage.

## Figures and Tables

**Table 1:** Antifeedant activities of lignans from stem bark of *Zanthoxylum armatum*.

Compound	Antifeedant Index (%, Mean ± SD)					EC_50_ (ppm)
	25 ppm	74 ppm	222 ppm	667 ppm	2000 ppm	
Asarinin (**1**)	49.45 ± 3.78	55.51 ± 1.79	68.03 ± 4.22	70.72 ± 2.83	75.83 ± 3.69	25.64 ± 3.21
Fargesin (**2**)	40.65 ± 4.51	52.12 ± 2.31	60.71 ± 3.21	75.21 ± 1.39	81.96 ± 2.93	63.24 ± 4.83
Horsfieldin (**3**)	34.99 ± 5.32	48.91 ± 3.37	73.74 ± 3.04	73.79 ± 6.01	81.12 ± 3.33	68.39 ± 5.58
Eudesmin (**4**)	35.73 ± 4.37	41.28 ± 3.07	43.57 ± 2.16	55.97 ± 3.46	69.82 ± 3.56	245.72 ± 3.74
Planispine (**5**)	-	-	-	-	-	-
Planispine B (**6**)	29.76 ± 3.16	45.95 ± 5.11	47.79 ± 3.27	58.38 ± 3.17	68.64 ± 5.23	219.84 ± 4.69
Yangambin (**7**)	37.80 ± 3.12	49.71 ± 2.61	50.82 ± 4.27	60.95 ± 3.26	66.96 ± 4.16	132.50 ± 3.61
Acuminatolide (**8**)	39.15 ± 1.21	41.36 ± 3.83	54.51 ± 2.18	77.35 ± 3.65	74.76 ± 3.93	102.12 ± 2.40
Salicifoliol (**9**)	6.82 ± 3.47	12.52 ± 2.15	24.78 ± 3.17	30.46 ± 5.23	39.06 ± 4.17	1069.20 ± 3.83
Magnolone (**10**)	38.93 ± 4.29	47.52 ± 1.91	62.16 ± 3.93	71.94 ± 3.19	74.14 ± 3.95	78.37 ± 3.44
Magnone A (**11**)	21.66 ± 3.91	28.99 ± 3.85	32.76 ± 4.33	43.53 ± 2.77	56.60 ± 5.06	1203.53 ± 3.95
Toosendanin *	32.32 ± 2.28	52.45 ± 3.27	69.52 ± 2.47	76.54 ± 3.62	86.27 ± 3.51	71.69 ± 3.13

* represents the positive control. The antifeedant index of the blank control is 0.
